# *In vitro* activity of gallium-protoporphyrin IX against *Leishmania major* and *Leishmania infantum*

**DOI:** 10.1128/spectrum.02118-25

**Published:** 2026-01-30

**Authors:** Sara Maestrini, Aurora Diotallevi, Sarah Hijazi, Sara Habouria, Emanuela Frangipani, Luca Galluzzi

**Affiliations:** 1 Department of Biomolecular Sciences, University of Urbino Carlo Bo 204212, Urbino (PU), Italy; 2 Laboratory of Transmission, Control and Immunobiology of Infection, Institut Pasteur de Tunis 37965, Tunis, Tunisia; University of Arkansas for Medical Sciences, Little Rock, Arkansas, USA

**Keywords:** *Leishmania*, GaPPIX, heme-mimetic, iron starvation, anti-parasitic drugs

## Abstract

*Leishmania* spp., the causative agents of leishmaniasis, pose significant global health threats, with visceral leishmaniasis being the most severe and fatal form. The increasing drug resistance in the treatment of leishmaniasis emphasizes the urgent need for novel therapeutic approaches. One promising strategy involves targeting essential cellular mechanisms such as nutrient acquisition, particularly iron, which is critical for energy metabolism and signal transduction. *Leishmania* spp. cannot synthesize heme *de novo* and rely on host-derived iron and heme for survival and pathogenicity. Gallium [Ga(III)] is an iron [Fe(III)]-mimetic molecule that has emerged as a promising antimicrobial agent, offering a novel approach to combat infections, mainly by replacing Fe(III) in redox enzymes thereby disrupting essential metabolic pathways and impairing microbial viability. In this study, the antiparasitic activity of Ga (III)-protoporphyrin IX (GaPPIX) was tested against *Leishmania major* and *Leishmania infantum*, both in promastigotes and intracellular amastigotes. Our results demonstrate that GaPPIX inhibits the viability of both species, specifically targeting the enzymatic activity of cytochrome *c* oxidase, with a higher sensitivity observed in *L. major*. The inhibitory effect is reversed by hemin, suggesting specificity for *Leishmania* heme-dependent cellular processes and a possible cytostatic action. Moreover, we found that GaPPIX can effectively synergize with miltefosine. This feature, coupled with its minimal toxicity toward human cells, makes GaPPIX a good candidate to be potentially developed as a novel anti-*Leismania* agent.

**IMPORTANCE:**

This study is significant as it addresses a critical challenge in leishmaniasis management, namely, the increasing incidence of drug resistance and toxicity, compounded by the scarcity of effective therapeutic options. We demonstrate that GaPPIX, a heme-mimetic compound, exhibits potent antiparasitic activity against both *Leishmania major* and *Leishmania infantum*, while displaying minimal cytotoxicity toward human cells, underscoring its potential as a safe and targeted therapeutic candidate. Importantly, the ability of GaPPIX to synergize with the first-line drug miltefosine highlights its translational relevance in combination therapies, which are essential for overcoming resistance and improving treatment efficacy. Collectively, these findings advance GaPPIX as a promising approach for the development of innovative therapeutics against a neglected but globally significant disease.

## INTRODUCTION

*Leishmania* spp. are protozoan parasites causing leishmaniasis, a disease ranging from cutaneous lesions to severe visceral forms. Visceral leishmaniasis, the deadliest type, is fatal in over 95% of untreated cases (1). The parasite is transmitted via sand fly vectors and develops intracellularly within macrophages. Leishmaniasis disproportionately affects vulnerable populations and is endemic in 98 countries across Europe, Africa, Asia, the Americas, and Oceania. Annually, over 30,000 visceral leishmaniasis cases and about one million cutaneous cases are reported globally (2). The rise of drug resistance in the treatment of leishmaniasis highlights the urgent need for novel therapies.

Targeting parasite nutrient acquisition, particularly iron, offers a promising approach for innovative anti-*Leishmania* agents. Iron is a key co-factor for numerous vital enzymes involved in essential biological functions like DNA synthesis and energy metabolism (3). Moreover, in *Leishmania*, many heme-containing proteins have been identified and characterized to be involved in various biological processes such as respiration, defense from oxidative stress, signal transduction, and sterol synthesis (4). *Leishmania* depends on host-derived iron and heme for survival and pathogenicity, as it lacks cytosolic iron-storage proteins and cannot synthesize heme *de novo*, possessing only partial heme biosynthetic pathways (4, 5). For that reason, the parasite employs sophisticated strategies to acquire these nutrients, including manipulating host iron storage and synthesizing transporters to uptake iron and heme from host phagolysosomes (6). For instance, a *Leishmania* iron transporter (LIT1), essential for parasite replication and virulence, has been identified in *Leishmania* genomes (7). Moreover, *Leishmania* heme response 1 (LHR1) has been found to facilitate heme transport and regulate intracellular heme levels in *Leishmania amazonensis*. Knockout of LHR1 impairs the parasite replication within macrophages and diminishes its virulence (8, 9). Another porphyrin transporter essential for the parasite, which has been shown to be involved in heme uptake, is LFLVCRb (10).

By leveraging these mechanisms, gallium [Ga(III)] can potentially substitute for iron in critical metabolic processes, such as cellular respiration. This substitution disrupts essential metabolic pathways and may result in cell death, as Ga(III) is unable to participate in redox reactions fundamental for these processes. Many Ga(III) formulations, such as Ga(III)-nitrate and Ga(III)-protoporphyrin (GaPPIX), have been developed (11) and have demonstrated efficacy against a broad spectrum of bacterial pathogens (12, 13). GaPPIX and hemin are both heme analogs, but while hemin may serve as a heme source, supporting various heme-dependent biological processes, GaPPIX can disrupt metabolic pathways in which heme is involved. Notably, GaPPIX showed a potent activity against *Haemophilus influenzae*, which lacks the ability to synthesize heme *de novo*, similarly to *Leishmania* (13). However, only one recent study from 2020 has investigated the activity of alkyl Ga(III) derivatives against *Leishmania major*, with IC_50_ values ranging from 1.11 μM to 13.4 μM (14). Given the reliance of *Leishmania* on external heme sources and the critical role of heme in their mitochondrial electron transport chain, it is plausible that GaPPIX could disrupt cellular respiration by impairing its function (15, 16).

In this study, the antiparasitic effect of GaPPIX was tested against *L. major* (a dermotropic species) and *Leishmania infantum* (a viscerotropic species) (17, 18). In fact, dermotropic and viscerotropic species may have adapted to different heme availability, depending on localization of host macrophages (dermis or spleen/liver for dermotropic and viscerotropic species, respectively) (19). We have shown that GaPPIX effectively inhibits the viability of both species, specifically inhibiting the enzymatic activity of cytochrome *c* oxidase (COX), an essential component of mitochondrial function, with minimal toxicity toward human cells. A stronger activity was observed in *L. major*, suggesting its higher susceptibility. In addition to its potential as a standalone treatment, GaPPIX was also tested in combination with the existing anti-*Leishmania* drug miltefosine (MILT).

## MATERIALS AND METHODS

### Parasite and cell cultures

*L. infantum* MHOM/TN/80/IPT1 (WHO international reference strain) was purchased from ATCC (ATCC 50134). *L. major* MHOM/TN/94/GLC94 (reference strain) was obtained from Pasteur Institute of Tunis. *L. infantum* and *L. major* promastigotes were cultured in Evans’ Modified Tobie’s Medium at 26°C–28°C. For viability tests, the parasites were resuspended in RPMI supplemented with peptone and yeast extract (RPMI-PY) medium as described by Castelli et al. (20), with a modification. The medium was supplemented with 5% heat-inactivated fetal bovine serum (FBS) instead of 10%, to minimize iron content. The human monocytic cell line THP-1 (ECACC 88081201) was cultured in RPMI-1640 medium supplemented with 10% FBS, 100 μg/mL streptomycin, and 100 U/L penicillin and maintained in a humidified incubator at 37°C and 5% CO_2_. Cells were treated for 72 h with 20 ng/mL phorbol myristic acid (PMA) to induce differentiation into macrophage-like cells. Toxicity tests were carried out in RPMI-1640 medium supplemented with 5% FBS, 100 μg/mL streptomycin, and 100 U/L penicillin. All culture reagents were purchased from Sigma-Aldrich.

Gallium Protoporphyrin IX Ga(III) Chloride (GaPPIX) (Frontier Scientific) was prepared as a 50 mM stock solution in dimethyl sulfoxide (DMSO) and stored at 4°C in the dark (16). Bovine hemin chloride (Sigma-Aldrich) was freshly prepared in 50 mM NaOH as a 15 mM stock solution. Miltefosine (Sigma-Aldrich) was prepared as a stock solution of 40 mM in ddH₂O and stored at −20°C.

### *L. infantum* and *L. major* promastigotes viability assay

To evaluate if reducing FBS content in RPMI-PY could affect parasite viability, late log/stationary phase *L. major* and *L. infantum* promastigotes were resuspended in RPMI-PY medium supplemented with 10% or 5% FBS at a density of 2.5 × 10^6^ parasites/mL in 96-well plates (100 μL/well) and incubated for 72 h at 26°C. The CellTiter 96 Aqueous Non-Radioactive Cell Proliferation Assay (Promega), which relies on the ability of viable cells to convert a soluble tetrazolium salt [3-(4.5-dimethylthiazol-2-yl)-5-(3-carboxymethoxy-phenyl)-2-(4-sulfophenyl)-2H-tetrazolium (MTS)] into a formazan product, was employed as reported previously (21). Briefly, 20 μL of MTS/phenazine methosulfate mixture (20:1 ratio) was added to 100 μL of cell culture and incubated at 26°C until formazan production was evident. Absorbance was then recorded using a Microplate Reader (Infinite F50 Plus, Tecan) at 492 nm.

The activity of GaPPIX was first investigated at 50, 10, and 2 μM for 72 h at 26°C to obtain a preliminary evaluation of the IC_50_ values. Subsequently, to calculate the specific IC_50_, *L. major* and *L. infantum* promastigotes were exposed to scalar dilutions (1:2 or 2:3) of GaPPIX at concentrations ranging from 0.08 to 10 μM for 72 h at 26°C. A negative control with untreated parasites and a positive control consisting of the parasites treated with the anti-leishmanial drug MILT at concentrations ranging from 20 to 0.625 μM was included. Each condition was performed in duplicate. Promastigote viability was evaluated as described above, and the percentage of inhibition for each well was calculated. Controls containing only GaPPIX in the medium, at the same concentrations used for treatments, were included. The absorbance attributable to GaPPIX alone was subtracted from all experimental conditions to specifically evaluate the parasite response to the compound. The IC_50_ values were calculated using nonlinear regression curves in GraphPad Prism 8.0 (GraphPad Software, Inc., San Diego, CA, USA). The equation used for data fitting was *Y*= 100/(1 + 10∧((Log IC_50_ − *X*) × HillSlope)) (hillslope not constrained), where *X* represents the logarithm of concentration and *Y* is the normalized response.

### Evaluation of cytotoxicity on THP-1 cells

The cytotoxicity of GaPPIX was evaluated using THP-1 cells seeded at a density of 5 × 10^5^ cells/mL in 100 μL per well in a 96-well plate. Cells were treated for 72 h with 20 ng/mL PMA to induce differentiation into macrophage-like cells. After cell adhesion to the plate, GaPPIX was tested at different concentrations, obtained through 1:2 or 2:3 scalar dilutions of the molecule (ranging from 1 mM to 8 μM) for 72 h at 37°C. A vehicle control (DMSO) was included in each experiment, as well as a positive control (MILT) at concentrations ranging from 100 μM to 12.5 μM. Each condition was performed in duplicate. To evaluate cytotoxicity, the CellTiter 96 Aqueous Non-Radioactive Cell Proliferation Assay was used, as described above. Controls containing only GaPPIX in the medium, at the same concentrations used for treatments, were included. The half-maximal cytotoxic concentration (CC_₅₀_) of GaPPIX in mammalian cells was determined using non-linear regression curves in GraphPad Prism 8.0, as described above. The selectivity index (SI) of GaPPIX on *L. infantum* and *L. major* parasites was calculated as the ratio between their respective cytotoxicity on THP-1 (CC_50_, 72 h) and activity against promastigotes (IC_50_, 72 h).

### Hemin and GaPPIX competition test on *L. infantum* and *L. major* promastigotes

To evaluate the effect of hemin as a competitor of GaPPIX, late log/stationary phase *L. major* and *L. infantum* promastigotes were resuspended in RPMI-PY medium at a density of 2.5 × 10^6^ parasites/mL in 96-well plates (100 μL/well). *L. major* promastigotes were treated with scalar dilutions (1:2 or 2:3) of hemin ranging from 10 to 0.625 μM in the presence of 1 μM GaPPIX, while *L. infantum* promastigotes were treated with scalar dilutions (1:2 or 2:3) of hemin ranging from 50 to 0.75 μM in the presence of 5 μM GaPPIX. Both experiments were conducted for 72 h at 26°C. A negative control (untreated parasites) and a positive control (MILT) were included in the experiments. MILT was tested at 5 μM for both species, while hemin concentrations ranged from 0.3 to 50 μM. Each experimental condition was carried out in triplicate. Promastigote’s viability was evaluated using the CellTiter 96 Aqueous Non-Radioactive Cell Proliferation Assay, as described above. To specifically investigate the parasite response to the competition tests, control samples containing only GaPPIX and hemin in the medium, at the same concentrations used for treatments, were included. The absorbance attributable to the two molecules alone was subtracted from all experimental conditions to evaluate the parasite response to the treatments.

### GaPPIX treatment and hemin recovery test on *L. infantum* and *L. major* promastigotes

To evaluate a potential reversibility of the inhibitory action of GaPPIX against the parasites, late log/stationary phase *L. major* and *L. infantum* promastigotes were resuspended in RPMI-PY medium at a density of 5 × 10^6^ parasites/mL in 96-well plates (100 μL/well). Parasites were treated with GaPPIX or MILT at a final concentration of 10 and 5 μM, respectively, for 72 h at 26°C. After 72 h of treatment, further 100 μL fresh medium containing hemin was added, resulting in final hemin concentrations of 0, 5, 10, 20, 30, and 40 μM (for GaPPIX treatment) or 0, 15, 20, 25, 40, and 50 μM (for MILT treatment), and incubated for further 72 h. Experiments included a negative control consisting of untreated parasites. Each experimental condition was carried out in triplicate. To evaluate promastigote viability, the CellTiter 96 Aqueous Non-Radioactive Cell Proliferation Assay was employed, as described above. Controls containing only GaPPIX and hemin in the medium, at the same concentrations used in the treatments, were included. The absorbance resulting from the two molecules alone was subtracted from all the experimental conditions to specifically assess the parasite response to the treatments.

### Anti-amastigotes activity of GaPPIX on *L. infantum* and *L. major* infected cells

The activity of GaPPIX against intracellular *L. infantum* and *L. major* amastigotes was evaluated in THP-1-infected cells. THP-1 cells were seeded at a density of 5 × 10^5^ cells/mL in a 96- well plate (100 μL/well) and treated with 20 ng/mL PMA for 72 h to induce differentiation into macrophage-like cells. After differentiation, cells were infected using a parasite-to-cell ratio of 10:1 for 24 h with *L. infantum* or *L. major* late log/stationary phase promastigotes, previously labeled with carboxyfluorescein succinimidyl ester (CFSE) dye (eBioscience). Briefly, *Leishmania* promastigotes were washed twice with phosphate-buffered saline (PBS) to remove any trace of serum and resuspended at 1 × 10^7^ parasites/mL in pre-warmed PBS. CFSE was added at a final concentration of 10 μM, and promastigotes were incubated for 15 min at 37°C in the dark. The reaction was stopped by adding two volumes of cold complete medium (containing ≥10% serum) followed by incubation on ice for 5 min (22). Then, labeled parasites were washed three times with complete RPMI medium, the first wash using RPMI with 10% FBS and the subsequent two washes with RPMI containing 2% FBS, to limit iron availability. These labeled parasites were then used for infection. After 24 h of infection, cells were washed to remove free parasites, and the infected cells were exposed to GaPPIX at concentrations of 6.25, 12.5, 25, 50, and 100 μM for 72 h. A negative control consisting of infected but untreated THP-1 cells was included in the experiments. To monitor the infection, cells were washed, fixed with formaldehyde/methanol, and stained with Hoechst dye. Fluorescence microscopy was used to acquire images at 5× and 20× magnification with 494 nm excitation/521 nm emission (green) and 350 nm excitation/460 nm emission (blue) for CFSE and Hoechst staining, respectively. Automated cell counting of single-color images was performed with ImageJ software to monitor the rate of infection, which was determined by normalizing the amastigote count from each green image to the cell count from the corresponding blue image. The normalized infection rate for untreated infected cells was set as 100%. Each experiment has been performed at least twice.

### Measurement of COX activity in *L. infantum* and *L. major* promastigotes

COX activity was tested using the artificial electron donor N,N,N′,N′-tetramethyl-p-phenylene diamine (TMPD) (Fluka), freshly prepared as a stock solution of 0.54 M in ddH_2_O and used at a final concentration of 5 mM. Late log/stationary phase *L. major* and *L. infantum* promastigotes were resuspended in RPMI-PY medium at a density of 2.5 × 10^6^ parasites/mL and treated for 72 h with GaPPIX and/or hemin. In particular, *L. major* promastigotes were treated with 1 µM GaPPIX or 1 µM GaPPIX + 10 µM hemin, while *L. infantum* promastigotes were treated with 5 µM GaPPIX or 5 µM GaPPIX + 50 µM hemin. Untreated parasites were included as a negative control. After 72 h of treatment, *L. major* and *L. infantum* parasites were permeabilized using 20 µM digitonin (Santa Cruz Biotechnology). Permeabilization was directly carried out in RPMI-PY for 10 min at room temperature. After incubation, digitonin-permeabilized parasites were centrifuged at 670 × *g* for 10 min at 25°C and resuspended in 33 mM potassium phosphate buffer (KPi, pH 7.0). Then, parasites were seeded at a density of 3 × 10^7^ parasites/mL in 96-well plates (100 μL/well). The reaction was started by the addition of 10 µL of a 0.54 M TMPD solution to each well. The rate of TMPD oxidation was recorded spectrophotometrically at 520 nm for 8 min at 25 °C. Results were expressed as µmol TMPD oxidized/min/3 × 10^6^ parasites using 6.1 as the millimolar extinction coefficient of TMPD (16).

### Monitoring expression of COXIV and glycosomal GAPDH in *L. infantum* and *L. major*

The expression of COX subunit IV (*COXIV*) and *gGAPDH* genes was investigated in *L. major* and *L. infantum* promastigotes to gain insight into the different GaPPIX susceptibilities between the two species. To this end, total RNA was extracted from 20 × 10^6^ late log/stationary phase *L. major* and *L. infantum* promastigotes, directly lysed with 700 μL of QIAzol Lysis Reagent (Qiagen, Hilden, Germany). RNA extraction was performed with the miRNeasy Mini Kit (Qiagen, Hilden, Germany) following the manufacturer’s instructions. RNA was quantified using the Qubit 4 Fluorometer and the RNA HS Assay (Thermo Fisher Scientific, Waltham, MA, USA). The cDNA was synthesized from 500 ng total RNA using PrimeScript RT Master Mix (Perfect Real Time) (Takara Bio Inc.) according to the manufacturer’s instructions. The quantitative PCR was performed using 1 µL cDNA as template in 20 μL reaction mixture containing TB Green Premix Ex Taq II (Tli RNaseH Plus) Mastermix (Takara Bio Europe, France) and primers (200 nM) (Table S1). Mixtures were incubated at 95°C for 10 min, followed by 40 cycles at 95°C for 10 s, and 60°C for 50 s. The reactions were carried out in duplicate using a Rotorgene Q (Qiagen). Fold changes were calculated by relative quantification using the ΔΔCt method (23). Data were normalized using 60S ribosomal protein L30 as reference gene, and the relative gene expression was set to one for *L. infantum* samples.

### Drug combination assay on promastigotes

The combinatory effect of GaPPIX and MILT was evaluated against *Leishmania* promastigotes, using the checkerboard method following the protocol described by Principe et al. (24). Drugs were diluted in RPMI-PY and concentrations were selected to include a range that encompassed the previously determined IC_₅₀_ values for each compound. In each well of the resulting drug combination matrix, 100 μL of RPMI-PY containing approximately 5 × 10^6^ promastigotes/mL was aliquoted to obtain 200 μL final volume. Untreated *Leishmania* promastigotes and medium without parasites and drugs were included in each plate, as positive and negative controls, respectively. Then, plates were covered and incubated at 26°C for 72 h, after which MTS was added, as previously described, and plates were incubated for further 4 h. Absorbance at 492 nm was measured in a microplate reader (Infinite F50 Plus, Tecan). Control samples containing only GaPPIX in the medium, at the same concentrations used for treatments, were included, and the absorbance resulting from the compound alone was subtracted from all the experimental conditions to specifically consider the parasite response to the combinatory treatment of GaPPIX and MILT. MILT activity on parasites in the presence of increasing GaPPIX concentrations was evaluated through dose-response curves using GraphPad Prism 8.0, as described above. Drug interactions were analyzed using the CompuSyn software (ComboSyn Inc., Paramus, NJ, USA) (25) to evaluate drug synergism. The combination index (CI) values were calculated using the drug combination at non-constant ratio. Synergistic, additive, or antagonistic effects were established based on CI values: a CI < 0.9 indicates synergy, CI values between 0.9 and 1.10 suggest an additive effect, and a CI > 1.1 indicates antagonism. The dose reduction index (DRI) was calculated to estimate the fold reduction in dose when drugs were used in combination compared to the dose of each single drug.

### Statistical analysis

The evaluation of GaPPIX IC_50_ and CC_50_ values in *Leishmania* promastigotes and THP-1 cells, respectively, was performed using non-linear regression curves in GraphPad Prism 8.0 and expressed as means and 95% confidence interval (Conf. Int.).

The one-way ANOVA followed by Dunnett’s multiple comparisons test was used for comparisons involving three or more conditions. Unpaired *t*-test with Welch’s correction was used for comparisons between two conditions. A *P*-value < 0.05 was considered statistically significant. All statistical tests were performed in GraphPad Prism software 8.0.

## RESULTS

### GaPPIX shows an anti-parasitic activity against *L. infantum* and *L. major* promastigotes

The viability of *Leishmania* promastigotes was evaluated in RPMI-PY supplemented with 10% or 5% FBS, as described in the Materials and Methods section. Since the different FBS content did not significantly affect *L. major* and *L. infantum* promastigotes viability (Fig. S1), 5% FBS was used in all subsequent experiments to reduce the iron content in the culture medium. The activity against each *Leishmania* species was initially evaluated at three concentrations (50, 10, and 2 µM) to obtain a preliminary indication of the IC_50_. Based on these initial results, which indicated an IC_50_ value below 5 µM (data not shown), a dose response curve with GaPPIX concentrations ranging from 0.08 to 10 µM was performed. IC_50_ values of GaPPIX on *L. major* and *L. infantum* promastigotes were 0.6 µM (95% Conf. Int. = 0.5–0.7 µM) and 4.0 µM (95% Conf. Int. = 3.3–4.8 µM), respectively (Table 1). The dose-response curves of GaPPIX, compared to MILT, are shown in Fig. S2 and represent the percentage of parasite growth inhibition at increasing GaPPIX concentrations. Notably, *L. major* promastigotes were more susceptible to GaPPIX than *L. infantum* promastigotes. Furthermore, the IC_50_ of GaPPIX for *L. infantum* was close to that of MILT, highlighting comparable efficacy against this species. In contrast, GaPPIX exhibited a significantly lower IC_50_ than MILT against *L. major*, indicating that GaPPIX has superior anti-parasitic activity against this species (Table 1). Of note, the growth inhibition never reached 100%, even at the highest concentrations of GaPPIX tested.

**TABLE 1:** GaPPIX and MILT potency on *L. major* and *L. infantum* promastigotes and cytotoxicity on THP-1 cells

	THP1	*L. major*		*L. infantum*	
	CC_50_ µM^*a*^	IC_50_ µM^*a*^	SI^*b*^	IC_50_ µM^*a*^	SI^*b*^
GaPPIX	199.9 (184.8 to 215.8)	0.6 (0.5 to 0.73)	330.7	4.0 (3.3 to 4.8)	50.5
MILT	36.2 (33.4 to 39.4)	1.9 (1.8 to 2.1)	19.1	2.8 (2.5 to 3.2)	12.9

^*a*^ IC_50_ and CC_50_ values are reported as mean and 95% Conf. Int.

^*b*^ SI indicates the selectivity index.

### GaPPIX shows low cytotoxicity on THP-1 cells

The dose-response curve of GaPPIX on THP-1 cells was performed to determine the CC_50_ value, as described in the Materials and Methods section. The resulting CC_50_ value was 199.9 µM (Table 1), which is higher than that of MILT (36.2 µM) (95% Conf. Int. = 33.4–39.4 µM). The dose-response curves of GaPPIX and MILT on THP-1 cells are shown in Fig. S3. Therefore, the molecule displays very low toxicity toward THP-1 cells, as previously shown in other cell types (e.g., HEK293 and HaCaT) (26), resulting in a great SI for both *L. major* and *L. infantum* promastigotes (Table 1).

### Hemin can antagonize the inhibitory activity of GaPPIX on *Leishmania* promastigotes

Hemin (a PPIX containing Fe(III) with a coordinating chloride ligand) and GaPPIX (a complex of Ga(III) with PPIX) are heme analogs that similarly interact within biological pathways. We used hemin as a competitor of GaPPIX to get insights into its mechanism of action in *Leishmania*. In particular, to confirm the specificity of GaPPIX for *Leishmania* heme uptake systems, we investigated whether the growth-inhibitory effect observed for GaPPIX could be attenuated by the presence of hemin, which is known to enter cells by exploiting heme-uptake routes (15, 16, 27).

**Fig 1:**
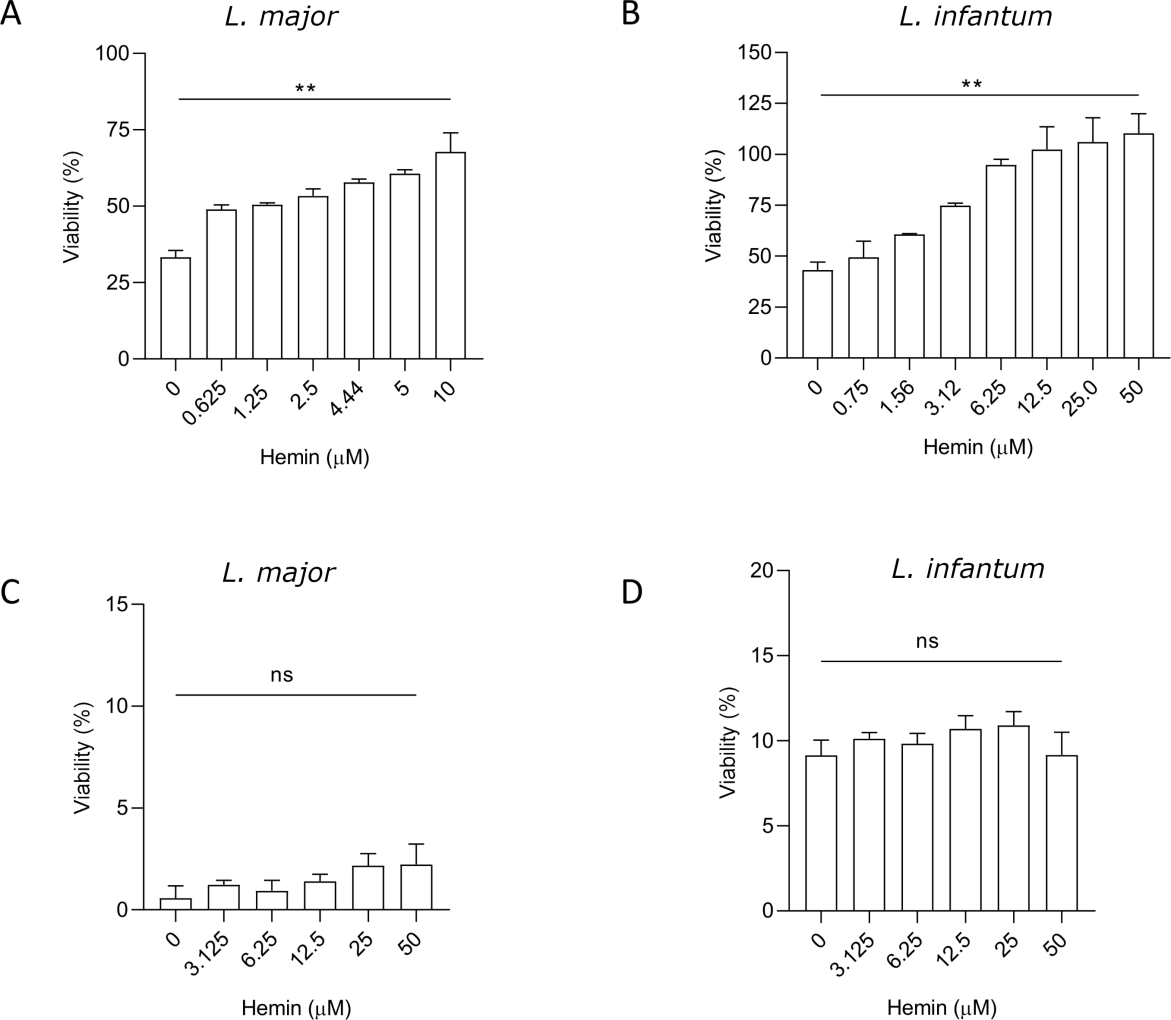
The effect of GaPPIX treatment is attenuated by hemin supplementation. *L. major* (**A**) and *L. infantum* (**B**) promastigotes were treated for 72 h with 1 and 5 µM GaPPIX, respectively, in the presence of increasing concentrations of hemin, as indicated in the panels. Parasite viability (measured using MTS assay, as described in the Materials and Methods section) increased proportionally with increasing hemin concentration. Hemin supplementation did not affect the drug’s effect on parasite viability in both *L. major* (**C**) and *L. infantum* (**D**) promastigotes treated with MILT. Data are represented as the mean ± SD. Statistical analysis was performed using one-way ANOVA with Dunnett’s multiple comparisons test. **P ≤ 0.01; not significant (ns) indicates *P*-value > 0.05.

Results showed that the viability of both *L. major* and *L. infantum* promastigotes was higher in the presence of hemin compared to the condition where only GaPPIX was used (Fig. 1A and B). Specifically, parasite vitality increased in a dose-dependent manner with increasing hemin concentration (up to 10 times the GaPPIX concentration). This dose-dependent effect was less pronounced on *L. major* promastigotes than in *L. infantum* promastigotes. In contrast, when MILT was used instead of GaPPIX, the presence of hemin (up to 10 times the MILT concentration) did not alter the drug’s effect on parasite viability (Fig. 1C and D), thus confirming the specific hemin-competitive effect toward GaPPIX.

### GaPPIX exhibits a cytostatic effect on *Leishmania* promastigotes

**Fig 2:**
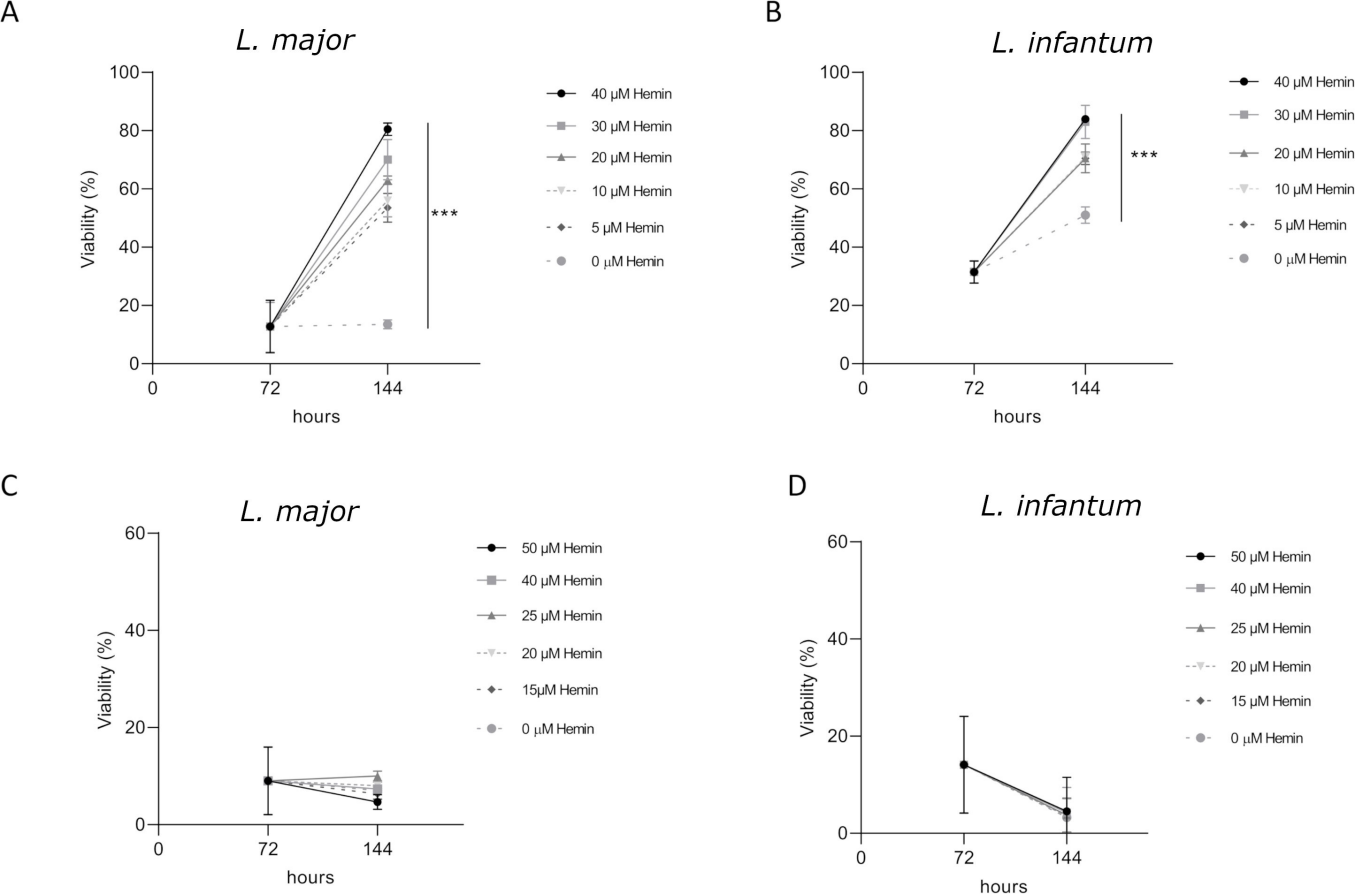
The effect of GaPPIX treatment can be reversed by the addition of hemin. After 72 h treatment with GaPPIX 10 µM, hemin was added, and cells were incubated for further 72 h. Cell viability was measured by MTS assay on *L. major* promastigotes (**A**) and *L. infantum* promastigotes (**B**). Following hemin supplementation, the vitality of the promastigotes increased in a dose-dependent manner. The hemin supplementation after MILT treatment did not show any significant effect in both *L. major* promastigotes (**C**) and *L. infantum* promastigotes (**D**) viability. Data are represented as the mean ± SD. Statistical analysis was performed using one-way ANOVA with Dunnett’s multiple comparisons test. ***P < 0.001.

To further investigate the effect of hemin on GaPPIX treatment and evaluate a potential reversibility of the inhibitory action of GaPPIX against *Leishmania*, an additional experiment was conducted. In this setup, GaPPIX was first added alone as described in the Materials and Methods section, and after 72 h, hemin was supplemented at different concentrations. When hemin was added 72 h after the initial GaPPIX treatment, the viability of *L. major* and *L. infantum* promastigotes increased in a dose-dependent manner, suggesting that GaPPIX may have a cytostatic (reversible) effect within the concentration range tested (Fig. 2A and B). On the contrary, when cells were treated with MILT, the hemin supplementation had no effect on cell viability (Fig. 2C and D).

### Anti-amastigotes activity on *L. infantum* and *L. major*-infected cells

**Fig 3:**
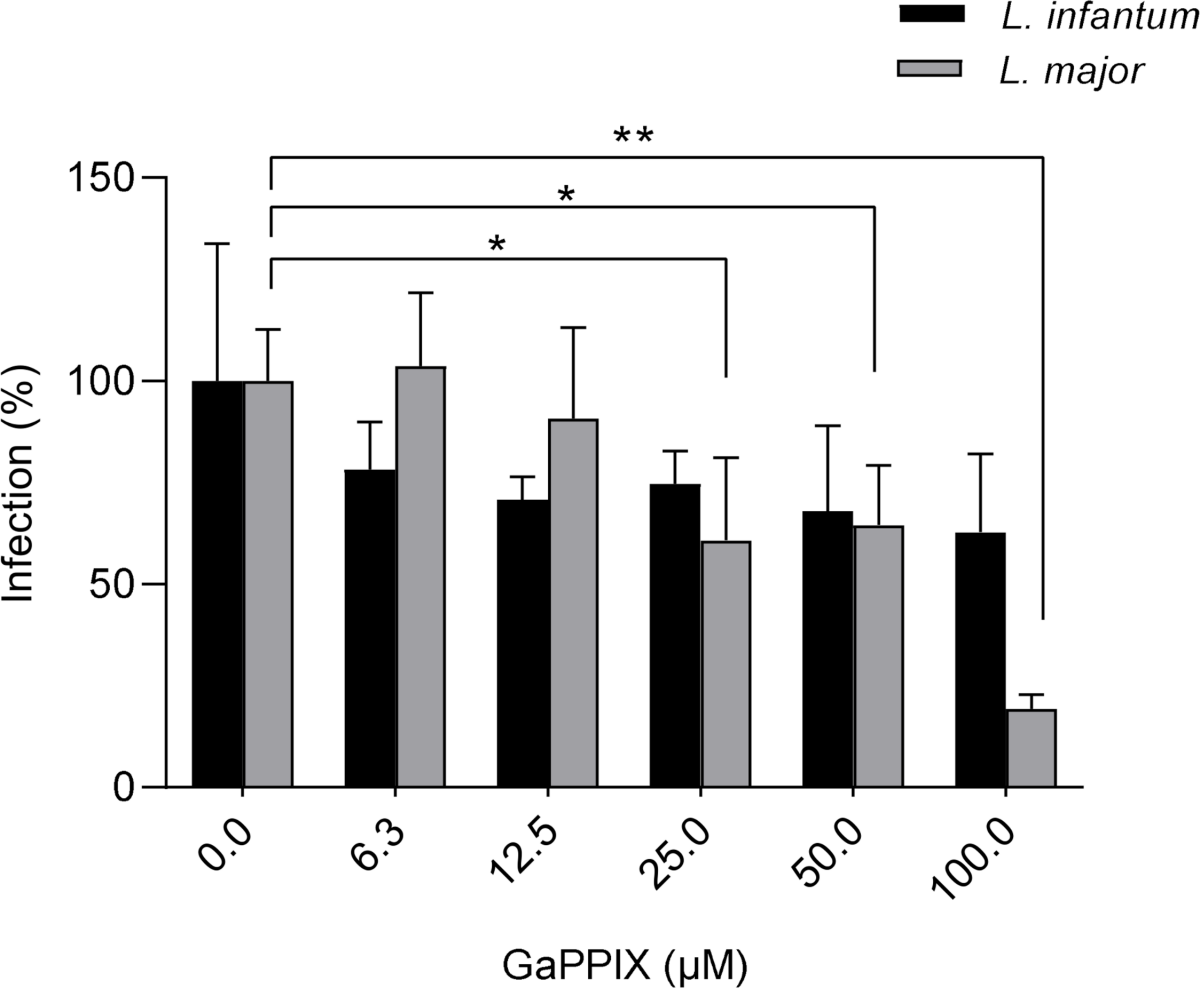
Effect of GaPPIX on intracellular *L. major* and *L. infantum* amastigotes. THP-1 cells were infected for 24 h at 37°C; then GaPPIX was added, and the efficacy on intracellular amastigotes was calculated after 72 h of treatment. Data are presented as the mean ± SD. Statistical analysis was performed using one-way ANOVA with Dunnett’s multiple comparisons test. *P < 0.05 and **P < 0.01.

Since GaPPIX was found to be effective against both *L. major* and *L. infantum* promastigotes, it was further tested on intracellular amastigotes in an *in vitro* infection model. Human monocytic THP-1 cell line was infected with *L. major* and *L. infantum* parasites, and infection was quantified as described in the Materials and Methods section. The analysis showed that treatment with GaPPIX reduced the infection rate in a dose-dependent manner (Fig. 3 and Fig. S4). Notably, in *L. major*-infected cells, the percentage of infection was significantly reduced at concentrations ≥25 µM. In contrast, despite an average reduction in infection rate, the GaPPIX treatment did not show any significant effect in *L. infantum*-infected cells. Therefore, *L. major* amastigotes appeared more susceptible to GaPPIX than *L. infantum* amastigotes, in line with what was observed in promastigotes. A non-linear regression analysis indicated a 50% reduction in infection rate at GaPPIX concentration of about 50 µM.

### GaPPIX targets COX in *L. infantum* and *L. major* promastigotes

**Fig 4:**
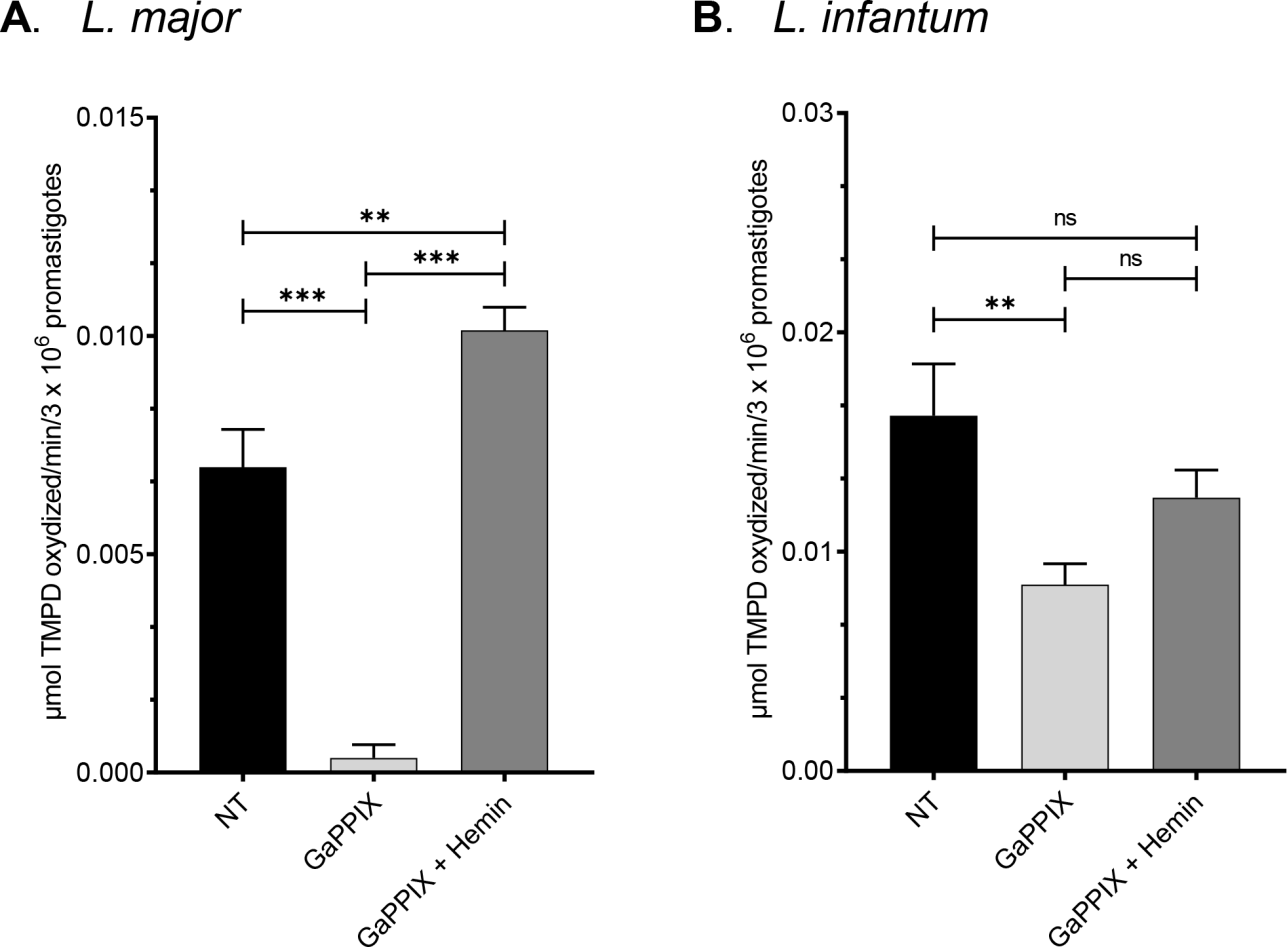
GaPPIX inhibits COX in *L. major* promastigotes and *L. infantum* promastigotes. (**A**) TMPD oxidase activity on whole *L. major* promastigotes untreated or treated with 1 µM GaPPIX or 1 µM GaPPIX + 10 µM hemin. (**B**) TMPD oxidase activity on whole *L. infantum* promastigotes untreated or treated with 5 µM GaPPIX or 5 µM GaPPIX + 50 µM hemin. Activity is expressed as μmol TMPD oxidized/min/3 × 10^6^ promastigotes at pH 7.0 at 25°C. Each value is the mean of three replicates ± SD. Statistical analysis was performed using one-way ANOVA with Dunnett’s multiple comparisons test. Differences among treatments and NT (non-treated) controls, as well as between treatments, were considered statistically significant (**P < 0.01 and ***P < 0.001); not significant (ns) indicates *P*-value > 0.05.

GaPPIX has been shown to be effective against a broad range of pathogenic bacteria, by targeting metabolic pathways that depend on heme as the enzymatic cofactor, including cellular respiration (15, 16). Given its effectiveness against *L. major* and *L. infantum* parasites and the crucial role of heme in *Leishmania* metabolism, we investigated whether GaPPIX could target the COX enzyme in the respiratory chain of the parasite, using the artificial electron donor TMPD. Specifically, the oxidation of TMPD to a blue indophenol compound reflects electron flow to the cytochrome *c* terminal oxidases, and this oxidation can be detected at 520 nm. As shown in Fig. 4, the micromoles of TMPD oxidized per minute by 3 × 10^6^ *L. major* promastigotes treated with 1 µM GaPPIX are nearly undetectable compared to the untreated condition and to the treatment with both GaPPIX and hemin. This suggests that COX is sensitive to GaPPIX and that hemin treatment restores its activity in this species.

A similar effect in reducing the rate of TMPD oxidation following GaPPIX treatment was observed in *L. infantum*, despite less pronounced (Fig. 4). The enzymatic activity of COX in *L. infantum* promastigotes treated with GaPPIX was not completely abolished, but it was reduced approximately by 50% compared to the untreated control. However, hemin treatment restored the activity of the enzyme to levels comparable to the untreated control, even though its effect did not differ significantly from that of GaPPIX alone.

Interestingly, we found that COX activity in untreated *L. infantum* was almost doubled compared to untreated *L. major* (Fig. 4). Since the sequences of COX subunits between the two species shared ≥97% identity, we hypothesized that the observed differences in activity may be due to variations in the expression of the *COX* genes between these two species. To this end, the expression of *COXIV* (encoding the subunit IV of COX) was monitored in both species. Unexpectedly, we found that *COXIV* gene was near sixfold more expressed in *L. major* than in *L. infantum* (Fig. S5), allowing to exclude *COXIV* overexpression as part of the tolerance mechanism in *L. infantum*. On the other hand, the expression of *GAPDH*, a canonical housekeeping gene, which is also known for a heme chaperone function in mammalian cells (28), did not show a significant difference between *L. major* and *L. infantum* (Fig. S5).

### Combination effects of GaPPIX and MILT against *L. infantum* and *L. major* promastigotes

**Fig 5:**
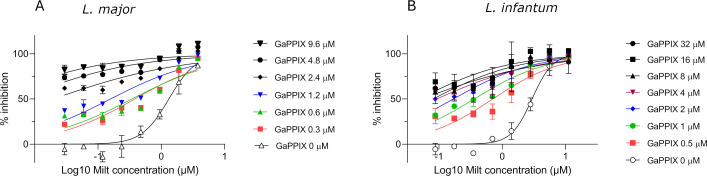
Increasing concentration of GaPPIX enhances the antileishmanial activity of MILT. Dose-response curves of MILT with GaPPIX co-treatment in *L. major* promastigotes (**A**) and *L. infantum* promastigotes (**B**), as obtained by the checkerboard method. The concentration of GaPPIX was constant as indicated for each curve, while that of MILT varied.

The drug interaction between GaPPIX and the antileishmanial drug MILT was investigated on *L. major* and *L. infantum* promastigotes using the drug combinations generated by the checkerboard method, as described above. The effect of MILT on parasites in the presence of increasing GaPPIX concentration was assessed through dose-response curves, demonstrating a positive effect in both species (Fig. 5A and B). To further characterize the interaction between the two molecules, the drug synergism analysis was performed as described in the Materials and Methods section.

In the treatment of *L. major* with different combinations of the two drugs, the analysis of the non-constant ratio combination of GaPPIX and MILT revealed a dose-dependent behavior (Table 2; Fig. S6). In fact, at high concentrations of GaPPIX (9.6–4.8 μM), the effect was mainly additive/synergic, whereas at intermediate and low concentrations of GaPPIX (2.4–0.30 μM), increased variability in the response was observed. Moreover, a marked synergistic behavior was observed when MILT was administered at low doses (0.03–0.95 μM), in combination with GaPPIX. In contrast, at medium to high concentrations of MILT (0.24–3.8 μM), the combinations exhibited antagonistic interactions. The treatment of *L. infantum* with GaPPIX and MILT combinations also showed a dose-dependent trend (Table 3; Fig. S6). At high doses of GaPPIX (32–8 μM) combined with very low MILT concentrations (0.09–0.35 μM), the interaction was predominantly antagonistic (CI > 1) but shifted progressively toward synergy (CI < 1) as MILT concentrations increased (≥0.70 μM). At intermediate GaPPIX concentrations (4–2 μM), all combinations exhibited a general synergy. At low GaPPIX concentrations (1–0.5 μM) and low doses of MILT, the interaction was predominantly antagonistic, whereas at intermediate and high doses of MILT, the combination shifted toward synergy.

**TABLE 2:** Effects of GaPPIX and MILT combinations on *L. major* promastigotes

Drug combination non-constant ratio (μM)		Effect^*a*^	CI^*b*^	Interaction	DRI^*c*^	
GaPPIX	MILT				GaPPIX	MILT
9.6	0.03	0.83 ± 0.04	1.05	Additive	0.97	47.64
9.6	0.06	0.85 ± 0.01	0.89	Synergic	1.18	26.00
9.6	0.12	0.84 ± 0.06	1.02	Additive	1.06	12.43
9.6	0.24	0.86 ± 0.02	0.96	Additive	1.24	6.66
9.6	0.48	0.86 ± 0.01	1.11	Additive	1.24	3.33
9.6	0.95	0.94 ± 0.03	0.61	Synergic	3.93	2.84
9.6	1.9	0.99 ± 0.03	0.09	Synergic	347.88	10.87
4.8	0.03	0.74 ± 0.02	0.92	Additive	1.12	37.13
4.8	0.06	0.75 ± 0.02	0.88	Synergic	1.21	19.22
4.8	0.12	0.77 ± 0.02	0.86	Synergic	1.32	10.01
4.8	0.24	0.80 ± 0.02	0.79	Synergic	1.65	5.54
4.8	0.48	0.80 ± 0.01	0.99	Additive	1.60	2.73
4.8	0.95	0.93 ± 0.02	0.56	Synergic	6.15	2.54
4.8	1.9	0.99 ± 0.03	0.09	Synergic	695.75	10.87
2.4	0.03	0.62 ± 0.01	0.86	Synergic	1.21	28.06
2.4	0.06	0.62 ± 0.02	0.90	Synergic	1.20	14.00
2.4	0.12	0.60 ± 0.04	1.06	Additive	1.10	6.71
2.4	0.24	0.69 ± 0.01	0.82	Synergic	1.74	4.13
2.4	0.48	0.73 ± 0.01	0.91	Synergic	2.14	2.27
2.4	0.95	0.84 ± 0.02	0.87	Synergic	4.29	1.58
2.4	1.9	0.95 ± 0.01	0.70	Synergic	18.79	1.54
2.4	3.8	0.99 ± 0.01	0.18	Synergic	1,391.51	5.44
1.2	0.03	0.44 ± 0.10	0.98	Additive	1.08	19.45
1.2	0.06	0.42 ± 0.08	1.11	Antagonist	0.99	9.38
1.2	0.12	0.44 ± 0.01	1.12	Antagonist	1.09	4.89
1.2	0.24	0.46 ± 0.02	1.23	Antagonist	1.19	2.54
1.2	0.48	0.50 ± 0.02	1.45	Antagonist	1.39	1.37
1.2	0.95	0.73 ± 0.01	1.11	Antagonist	4.26	1.15
1.2	1.9	0.90 ± 0.01	1.00	Additive	16.58	1.06
1.2	3.8	0.98 ± 0.02	0.79	Synergic	114.17	1.28
0.6	0.03	0.31 ± 0.02	0.90	Synergic	1.20	14.89
0.6	0.06	0.33 ± 0.02	0.90	Synergic	1.29	7.70
0.6	0.12	0.31 ± 0.04	1.11	Antagonist	1.19	3.71
0.6	0.24	0.36 ± 0.05	1.14	Antagonist	1.52	2.07
0.6	0.48	0.40 ± 0.02	1.44	Antagonist	1.82	1.12
0.6	0.95	0.59 ± 0.03	1.44	Antagonist	4.23	0.83
0.6	1.9	0.86 ± 0.01	1.23	Antagonist	20.18	0.85
0.6	3.8	0.95 ± 0.01	1.31	Antagonist	75.14	0.77
0.3	0.03	0.22 ± 0.01	0.79	Synergic	1.42	11.76
0.3	0.06	0.32 ± 0.01	0.54	Synergic	2.48	7.57
0.3	0.12	0.26 ± 0.05	0.87	Synergic	1.79	3.26
0.3	0.24	0.45 ± 0.07	0.62	Synergic	4.59	2.50
0.3	0.48	0.41 ± 0.01	1.15	Antagonist	3.70	1.13
0.3	0.95	0.61 ± 0.02	1.27	Antagonist	9.06	0.86
0.3	1.9	0.82 ± 0.02	1.39	Antagonist	29.65	0.74
0.3	3.8	0.95 ± 0.01	1.32	Antagonist	146.75	0.76

^*a*^ Growth inhibition measured by the MTS assay and normalized to untreated controls considering one as the upper limit for total inhibition (mean ± SD of two biological replicates), in the presence of combinations of GaPPIX and MILT.

^*b*^ Combination index (CI) values indicate the levels of drug interaction. CI < 0.90 = synergism, CI 0.90–1.10 = additive, CI > 1.10 = antagonism.

^*c*^ Dose reduction index (DRI), a measurement of how much drug dosage can be reduced in a combination while maintaining the same effect compared to the dosage of drug used individually.

**TABLE 3:** Effects of GaPPIX and MILT combinations on *L. infantum* promastigotes

Drug combination non-constant ratio (μM)		Effect^*a*^	CI^*b*^	Interaction	DRI^*c*^	
GaPPIX	MILT				GaPPIX	MILT
32	0.09	0.70 ± 0.05	1.56	Antagonist	0.65	46.32
32	0.16	0.70 ± 0.04	1.49	Antagonist	0.69	26.22
32	0.35	0.70 ± 0.04	1.61	Antagonist	0.66	11.67
32	0.70	0.76 ± 0.06	0.79	Synergic	1.56	6.69
32	1.40	0.93 ± 0.16	0.17	Synergic	79.92	6.27
32	2.80	0.98 ± 0.05	0.17	Synergic	3,922.27	5.83
32	5.60	0.99 ± 0.06	0.11	Synergic	6,335,849	9.45
16	0.09	0.69 ± 0.01	0.87	Synergic	1.17	45.59
16	0.18	0.66 ± 0.04	1.21	Antagonist	0.86	21.80
16	0.35	0.65 ± 0.01	1.45	Antagonist	0.74	10.64
16	0.70	0.73 ± 0.06	0.67	Synergic	1.96	6.22
16	1.40	0.91 ± 0.08	0.19	Synergic	72.38	5.52
16	2.80	0.99 ± 0.01	0.05	Synergic	12,670,000	18.89
8	0.09	0.58 ± 0.03	1.55	Antagonist	0.66	37.21
8	0.18	0.57 ± 0.02	1.70	Antagonist	0.61	18.49
8	0.35	0.60 ± 0.03	1.37	Antagonist	0.79	9.64
8	0.70	0.69 ± 0.04	0.62	Synergic	2.26	5.70
8	1.40	0.86 ± 0.06	0.25	Synergic	36.61	4.44
8	2.80	0.97 ± 0.02	0.23	Synergic	2,610.62	4.38
8	5.60	0.99 ± 0.01	0.29	Synergic	55,398.90	3.56
4	0.09	0.56 ± 0.06	0.91	Additive	1.13	36.33
4	0.18	0.59 ± 0.05	0.69	Synergic	1.56	19.24
4	0.35	0.62 ± 0.03	0.58	Synergic	2.09	10.07
4	0.70	0.62 ± 0.08	0.67	Synergic	2.13	5.05
4	1.40	0.77 ± 0.08	0.36	Synergic	14.23	3.42
4	2.80	0.92 ± 0.03	0.34	Synergic	411.84	2.92
4	5.60	0.99 ± 0.03	0.31	Synergic	54,839.70	3.18
4	11.20	0.97 ± 0.01	0.93	Additive	4,786.82	1.08
2	0.09	0.53 ± 0.03	0.68	Synergic	1.54	34.17
2	0.18	0.52 ± 0.04	0.75	Synergic	1.44	17.01
2	0.35	0.58 ± 0.02	0.50	Synergic	2.54	9.31
2	0.70	0.63 ± 0.03	0.41	Synergic	4.73	5.14
2	1.70	0.78 ± 0.06	0.38	Synergic	31.18	2.86
2	2.80	0.94 ± 0.01	0.30	Synergic	2,033.04	3.37
2	5.60	0.98 ± 0.01	0.35	Synergic	53,298.80	2.84
2	11.20	0.99 ± 0.01	0.61	Synergic	135,959.00	1.65
1	0.09	0.40 ± 0.11	1.32	Antagonist	0.78	27.46
1	0.18	0.42 ± 0.02	1.11	Antagonist	0.96	14.28
1	0.35	0.49 ± 0.07	0.60	Synergic	2.12	8.10
1	0.70	0.53 ± 0.03	0.56	Synergic	3.07	4.30
1	1.40	0.65 ± 0.01	0.46	Synergic	11.63	2.65
1	2.80	0.88 ± 0.01	0.42	Synergic	483.41	2.40
1	5.60	0.96 ± 0.04	0.51	Synergic	11,119.50	1.98
1	11.20	0.97 ± 0.03	0.85	Synergic	33,742.90	1.18
0.5	0.09	0.31 ± 0.09	1.87	Antagonist	0.55	23.25
0.5	0.18	0.29 ± 0.02	2.60	Antagonist	0.40	11.11
0.5	0.35	0.33 ± 0.03	1.53	Antagonist	0.73	6.12
0.5	0.70	0.36 ± 0.08	1.37	Antagonist	0.95	3.19
0.5	1.40	0.57 ± 0.11	0.54	Synergic	9.74	2.31
0.5	2.80	0.78 ± 0.06	0.58	Synergic	128.59	1.74
0.5	5.60	0.95 ± 0.03	0.58	Synergic	9,489.06	1.73
0.5	11.20	0.98 ± 0.02	0.82	Synergic	83,886.20	1.22

^*a*^ Growth inhibition measured by the MTS assay and normalized to untreated controls considering one as the upper limit for total inhibition (mean ± SD of two biological replicates), in the presence of combinations of GaPPIX and MILT.

^*b*^ Combination index (CI) values indicate the levels of drug interaction. CI < 0.90 = synergism, CI 0.90–1.10 = additive, CI > 1.10 = antagonism.

^*c*^ Dose reduction index (DRI), a measurement of how much drug dosage can be reduced in a combination while maintaining the same effect compared to the dosage of drug used individually.

Overall, DRI determination in both species revealed high values for MILT under multiple combinations, indicating a reduction in the dose required when combined with GaPPIX (Tables 2 and 3).

## DISCUSSION

Current chemotherapeutic interventions for leishmaniasis, such as MILT, pentavalent antimonials, and amphotericin B, are hindered by several drawbacks, including high toxicity, lengthy treatment courses, limited efficacy in some endemic regions, and the alarming emergence of drug-resistant *Leishmania* strains. These limitations underscore the urgent need for the development of novel, safe, and effective anti-leishmanial agents. Within the search for new antimicrobials, some of us have explored the possibility to interfere with microbial iron acquisition as a novel strategy to inhibit pathogen growth (12, 16, 29, 30). In particular, the use of the heme mimetic GaPPIX has been shown to interfere with several iron-dependent metabolic pathways in a wide range of bacterial pathogens, which might be relevant to *Leishmania* parasites as well (12, 13, 16). Given the reliance of *Leishmania* species on heme acquisition to support growth and virulence, due to their limited capacity for *de novo* synthesis (4, 8) and the experimental evidence that zinc porphyrin, when used in photodynamic therapy, is effective in reducing both promastigote and amastigote forms of *Leishmania braziliensis* (31), in this work, we have investigated the potential anti-parasite activity of GaPPIX on two representative species of *Leishmania* (i.e., *L. major* and *L. infantum*).

Interestingly, we found that GaPPIX exhibited excellent activity against the motile promastigote form of *L. major* and *L. infantum* and low toxicity on THP-1 cells, therefore showing a very good SI, much higher than the one calculated for MILT, in both parasite species tested (Table 1). Notably, GaPPIX anti-parasite activity was more pronounced in *L. major* than *L. infantum*, as observed in promastigotes and intracellular amastigotes, suggesting a greater susceptibility of *L. major*.

The different susceptibility to GaPPIX between *L. major* and *L. infantum* could also be due to differences in the heme (and its analogs) uptake, although dedicated GaPPIX uptake experiments are needed to validate this hypothesis. It has been previously shown that the uptake of another heme analog [Zn(II) Mesoporphyrin IX (ZnMP)] differs between different dermotropic or viscerotropic *Leishmania* species, being higher in *L. major* compared to *L. infantum* or *Leishmania donovani* (27). In fact, *Leishmania* parasites must be adapted to differential nutritional environments depending on cell tropism. Viscerotropic species (i.e., *L. infantum*) replicate in macrophages inside the liver and spleen, where these cells are removing senescent erythrocytes from circulation and are recycling hemoglobin and heme (19). The availability of recycled hemoglobin within the infected macrophages provides an important source of heme to support the parasite viability. On the other hand, *Leishmania* dermotropic species (i.e., *L. major*) predominantly infect macrophages resident in the dermis, exhibiting M2-like profiles consistent with their role in promoting tissue homeostasis and repair (32). Therefore, it is possible that dermotropic parasites have more limited access to heme, thus requiring a more efficient porphyrin uptake system. However, this should be further investigated since LHR1 and LFLVCRB show high similarity (>90% identity) in *L. infantum* and *L. major*; moreover, their expression level was not found to correlate with heme analog uptake efficiency (27).

Another aspect that may be involved in the different susceptibility to GaPPIX of *L. major* and *L. infantum* could be the activity of the COX complex. In fact, although the enzymatic activity of COX in promastigotes of both *Leishmania* species under investigation was significantly reduced by GaPPIX treatment, *L. major* promastigotes exhibited a lower basal COX activity and a higher susceptibility to GaPPIX compared to *L. infantum* promastigotes (Fig. 4). COX is one of the major hemoproteins involved in cellular respiration, and its inhibition has a direct and measurable impact on metabolic efficiency and cellular viability. Previous studies have shown that heme analogs, lacking redox capability, can interfere with the incorporation of functional heme into hemoproteins, including COX (16, 33). Although other hemoproteins may be affected by GaPPIX and the observed inhibition of COX may be only a part of the overall impact on the parasite, we consider this effect representative of GaPPIX’s potential action on other hemoproteins as well. Since the protein sequences of COX subunits are over 97% identical between the two species, we explored the hypothesis that the different activity and susceptibility to GaPPIX observed may depend on differences in the amount of COX complex in the mitochondrion. In fact, since one possible mechanism of resistance might rely upon overexpression of the drug target gene (34), a higher activity of COX in *L. infantum* might be contributing to the lower responsiveness to GaPPIX treatment, in terms of both COX activity and cell viability. To this end, we measured gene expression of the *COXIV* gene, finding a near sixfold overexpression in *L. major*, therefore allowing to exclude a low expression of *COXIV* as part of the sensitivity mechanism in *L. major*.

However, due to the lack of transcriptional regulation in *Leishmania* parasites, the relevance of transcript quantification as a possible and efficient prediction of protein levels can be criticized. In fact, a correlation between mRNA and protein levels was found for only <70% of *Leishmania* genes (35).

Heme is a hydrophobic cytotoxic macrocycle and is mainly bound to proteins that are essential for cell survival (e.g., P450, cytochromes). The rest of the heme is available for trafficking by not yet specified mechanisms (36). In *Leishmania*, heme distribution might involve the presence of heme carriers or chaperones to avoid its toxicity. Among them, G-quadruplex DNA sequences have been shown to sequester heme *in vivo*, protecting cells from the pathophysiological consequences of free heme (37). G-quadruplexes have been recently described in *Leishmania* (38); therefore, *Leishmania* G-quadruplexes could also sequester heme. Moreover, it is known that *Leishmania* can accumulate porphyrins in discrete vesicles called porphyrinosomes (39). Recently, glyceraldehyde-3-phosphate dehydrogenase (GAPDH) has been shown to play a heme-chaperone role in mammal cells, driving labile heme to its protein targets (28). In *Leishmania*, GAPDH is a glycosomal enzyme (gGAPDH). However, a cytosolic form (cGAPDH) exists in *L. infantum*, whereas cGAPDH has evolved into a pseudogene in *L. major* (40). The expression levels of *gGAPDH* gene in *L. infantum* and *L. major* promastigotes were not significantly different (Fig. S5); however, the presence of cGAPDH in *L. infantum* could contribute to explain, at least in part, the lower susceptibility of *L. infantum* to heme analog GaPPIX.

Although further research is needed to fully elucidate the molecular mechanisms of GaPPIX action in *Leishmania*, our findings suggest that GaPPIX could pave the way for a new class of metal-based anti-leishmanial agents, addressing a critical gap in the treatment of this neglected tropical disease. Moreover, in addition to its potential as a standalone treatment, GaPPIX may also serve as an effective co-treatment alongside existing anti-*Leishmania* drugs. Indeed, therapeutic failure due to the emerging drug resistance phenomenon, especially in endemic countries, remains a growing problem in the management of leishmaniasis. To this end, many studies have investigated the efficacy of combining anti-*Leishmania* agents with different mechanisms of action (41, 42). The advantages of combination therapy include an increase in therapeutic efficacy, a reduction in the dosage administered and associated toxicity, and a decrease in drug resistance development.

In this study, the drug interaction between GaPPIX and the antileishmanial drug MILT was investigated on *L. major* and *L. infantum* promastigotes using the drug combinations generated by the checkerboard method. Although the combination analysis of GaPPIX and MILT showed an alternation between conditions of synergy, antagonism, or additivity, suggesting that the dosage ratio represents a critical point of the combination efficacy, nearly all different combinations evidenced a DRI >1 for MILT, an effective drug characterized by considerable toxicity. In particular, a synergistic effect was more evident in the intermediate doses tested: the combinations of GaPPIX 2.4 µM with serial dilution 1:2 (3.8–0.03 µM) of MILT and of GaPPIX 2 µM with serial dilution 1:2 (11.2–0.09 µM) of MILT in *L. major* and *L. infantum*, respectively, revealed the best synergistic effect, suggesting that the concentration of the two drugs synergizes when appropriately combined.

In summary, GaPPIX could enhance the efficacy of current therapies by potentially reducing the required dosages, mitigating side effects, and delaying or preventing the development of drug resistance in *Leishmania* clinical isolates, a growing concern in endemic regions.

## SUPPLEMENTAL MATERIAL

Supplemental material spectrum.02118-25-s0001.pdf Table S1; Fig. S1 to S6.

ASM does not own the copyrights to Supplemental Material that may be linked to, or accessed through, an article. The authors have granted ASM a non-exclusive, world-wide license to publish the Supplemental Material files. Please contact the corresponding author directly for reuse.
