# Supplementary material for: In vitro activity of gallium-protoporphyrin IX against Leishmania major and Leishmania infantum
Source: Microbiol Spectr. 2026 Jan 30;14(3):e02118-25. doi: 10.1128/spectrum.02118-25 (PMC12955387; doi:10.1128/spectrum.02118-25)
Supplement: Supplemental material — Table S1; Fig. S1 to S6. [file spectrum.02118-25-s0001.pdf]

Table S1. Primers used in this study.

Primer name	Primer sequence 5'-3'	Product length (bp)	Target
L30_F	AAGACCCCCATCCACCACTA	102	60S ribosomal protein L30 (LmjF.35.0240; LINF_350007500)
L30_R	GTCCCCAACATTTGTCACGG		
gGAPDH_F	ACTGCAGAACAACCTACCCG	75	GAPDH glycosomal (LmjF.30.2970; LINF_300035000)
gGAPDH_R	GAGTAGCCCCACTCGTTGTC		
COX4_F	GAACGAGTTGGCTGAGGACA	87	cytochrome oxidase subunit IV (LmjF.12.0670; LINF_120011900)
COX4_R	TTAAGGTTCCAGCGCGTCTT		

Figure S1

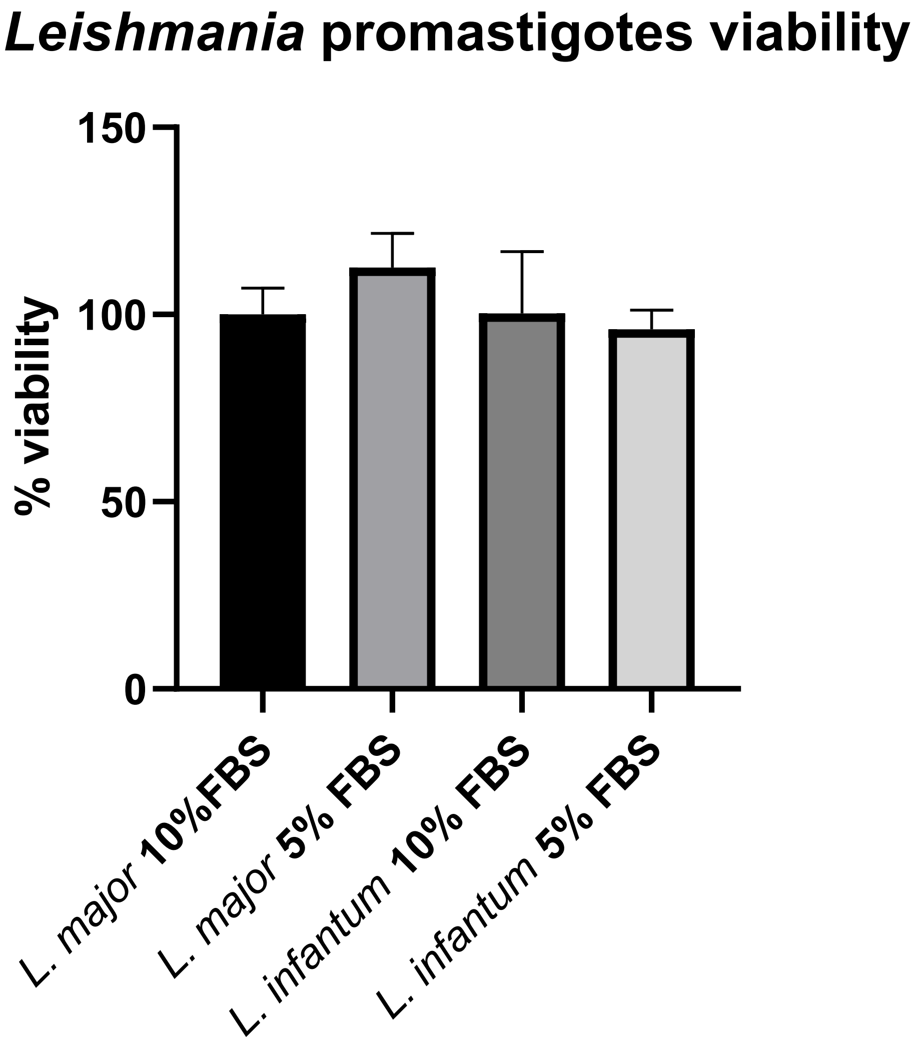

Fig S1. Viability of *L. major* and *L. infantum* promastigotes in RPMI-PY supplemented with 10% FBS or 5% FBS after 72h. Data are expressed as mean ± SD of two independent experiments performed in duplicate. Statistical analysis was performed using one-way ANOVA with Dunnett's multiple comparisons test. The viability was not significantly different (*P*-value > 0.05) in all condition tested.

Figure S2

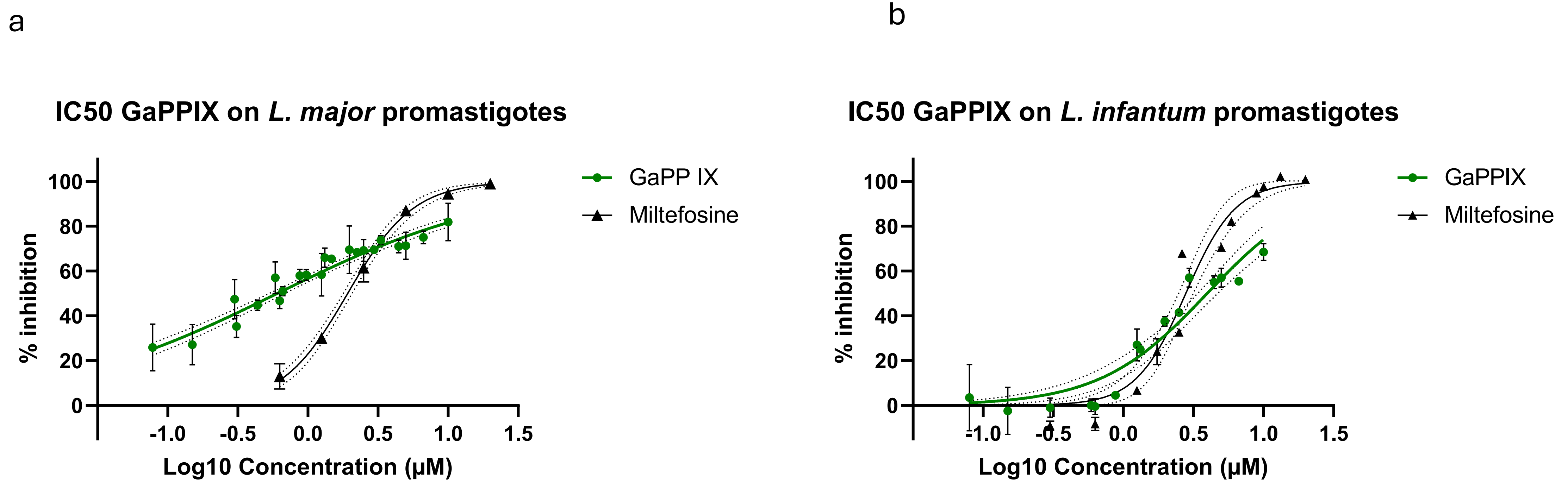

Fig S2. Dose-response curves determined using the MTS assay in *L. major* promastigotes (A) and *L. infantum* promastigotes (B) treated for 72 h with GaPPIX and miltefosine. Results were obtained from two independent experiments, each performed in duplicate. Data are expressed as mean ± SD.

Figure S3

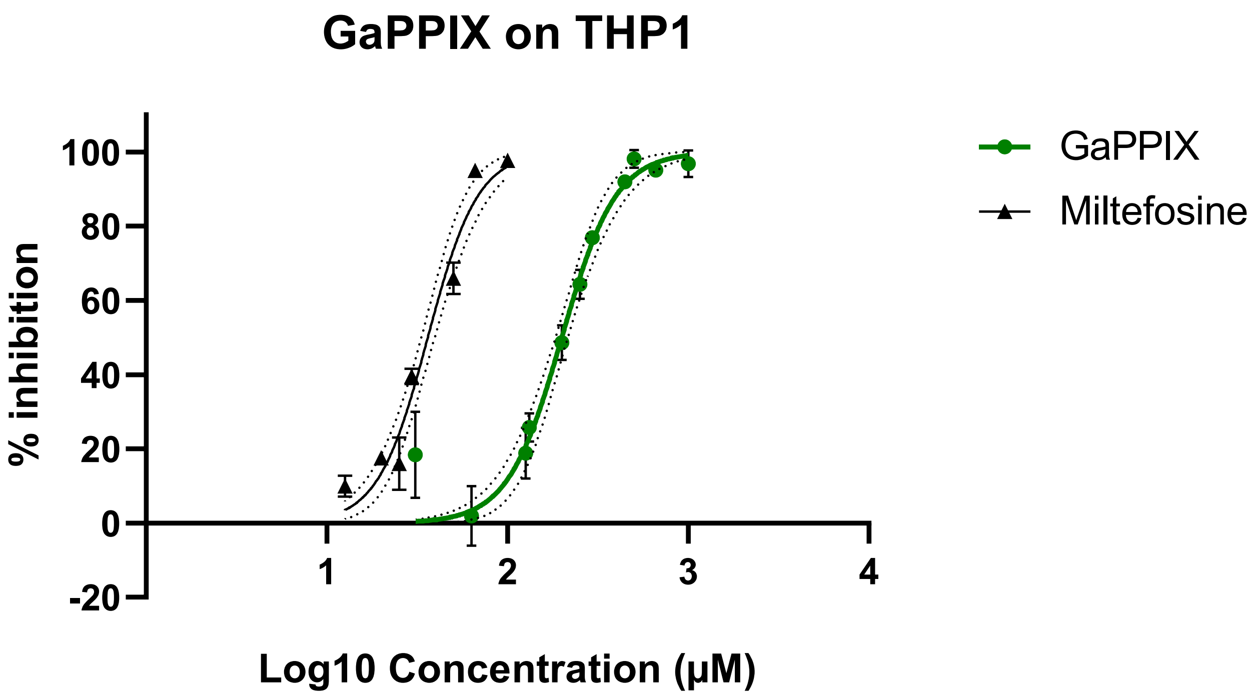

Fig S3. Dose-response curve determined using MTS assay of THP-1 cells treated for 72 h with GaPPIX and miltefosine. Results derived from two independent experiments, each performed in duplicate. Data are expressed as mean ± SD.

Figure S4

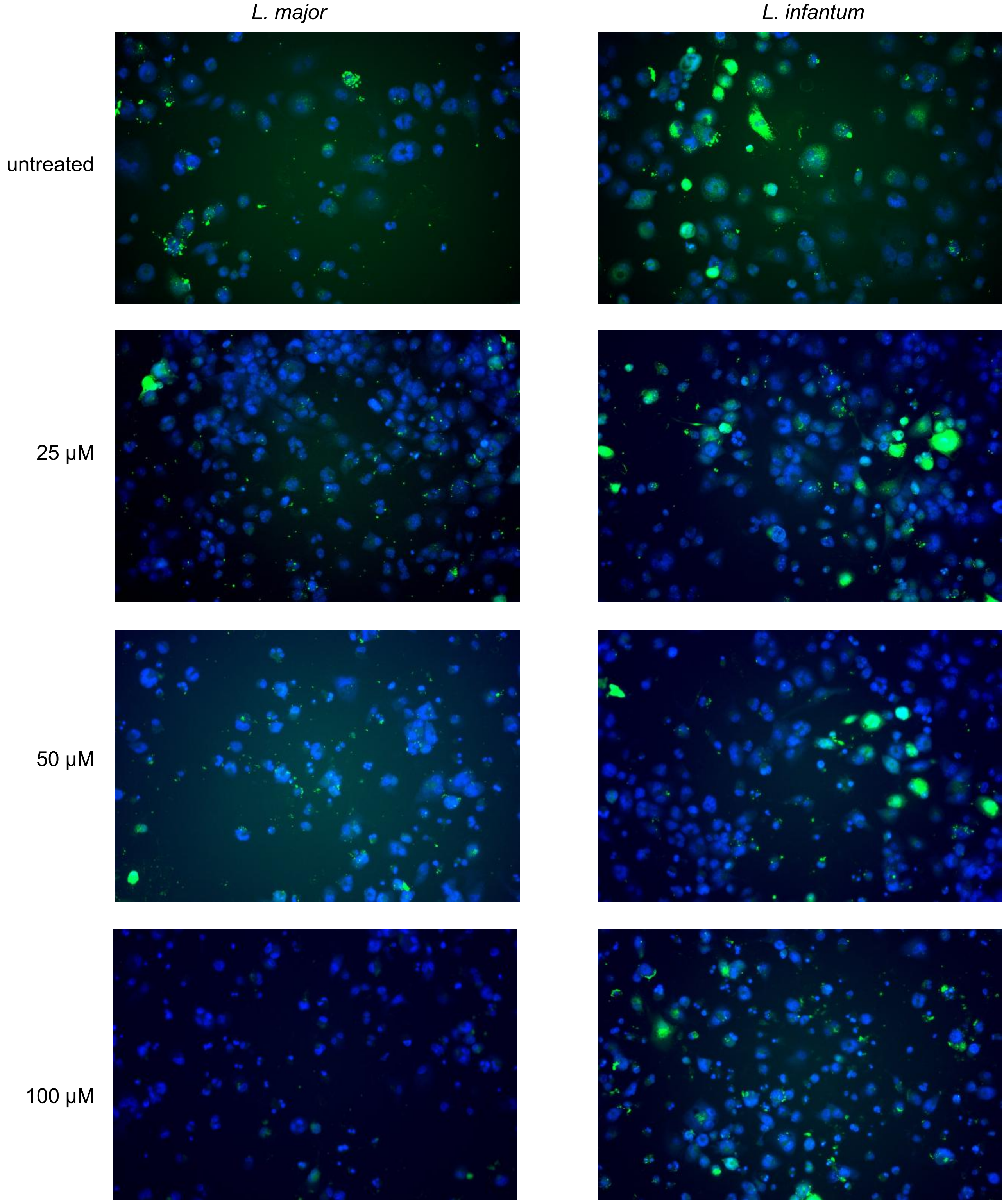

Fig S4. Representative images showing the effect of GaPPIX on intracellular *L. major* and *L. infantum* amastigotes. Differentiated THP-1 cells were infected for 24 h at 37 °C; then, GaPPIX was added and the efficacy on intracellular amastigotes was observed after 72 h of treatment. The images show THP-1 cells (stained with Hoechst dye) infected with *L. major* or *L. infantum* (stained with CFSE), untreated or treated with GaPPIX at the indicated concentrations.

Figure S5

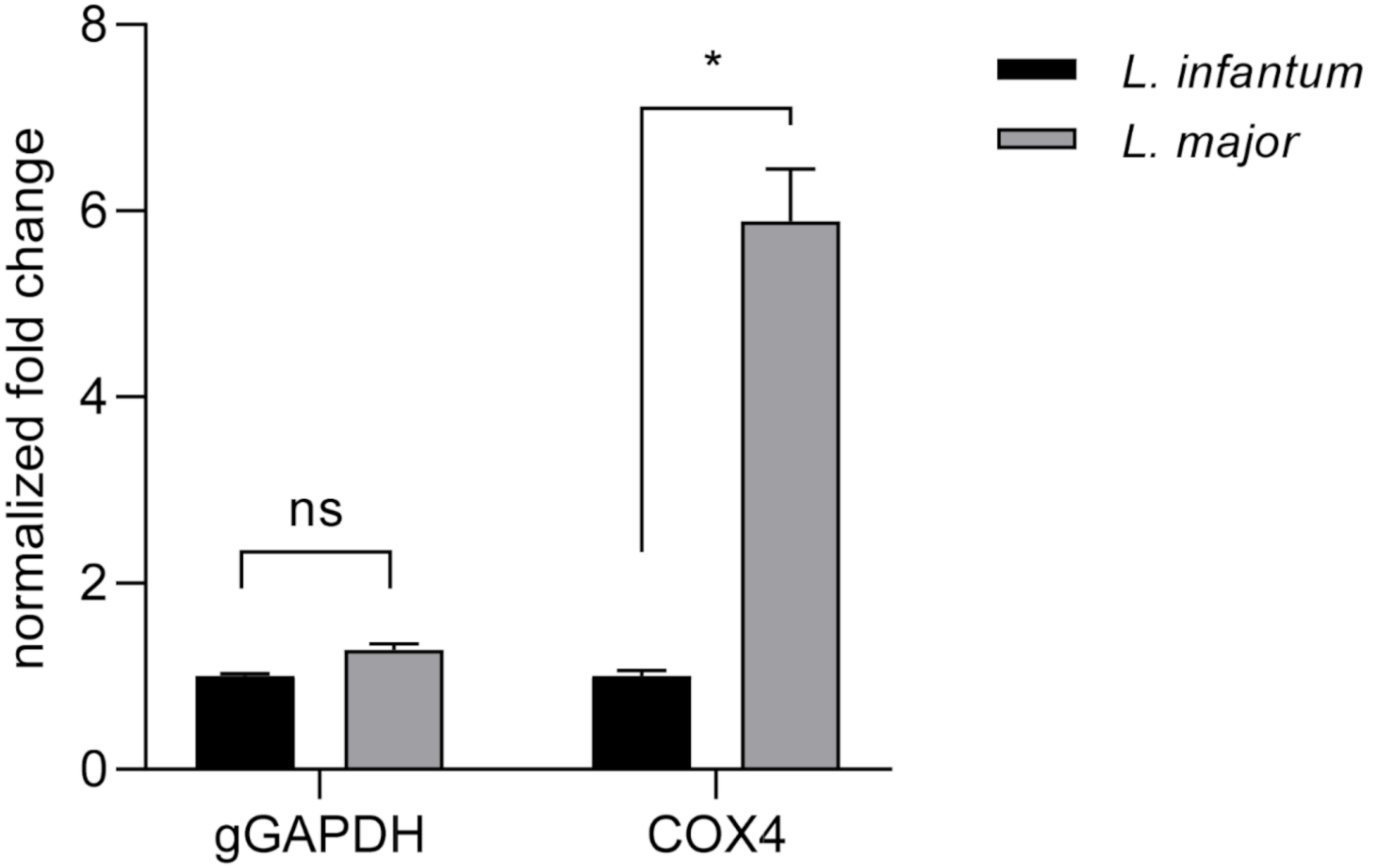

Figure S5. The expression of *COXIV* and *gGAPDH* was evaluated in *L. infantum* MHOM/TN/80/IPT1 and *L. major* MHOM/TN/94/GLC94 promastigotes. The expression of *COXIV* gene is higher in *L. major* promastigotes. Data were normalized using 60S ribosomal protein L30 as reference gene, and the relative gene expression was set to 1 for *L. infantum*. Statistical analysis was performed using Unpaired t test with Welch's correction. *$p<0.05$; not significant (ns) indicate a p-value > 0.05.

Figure S6

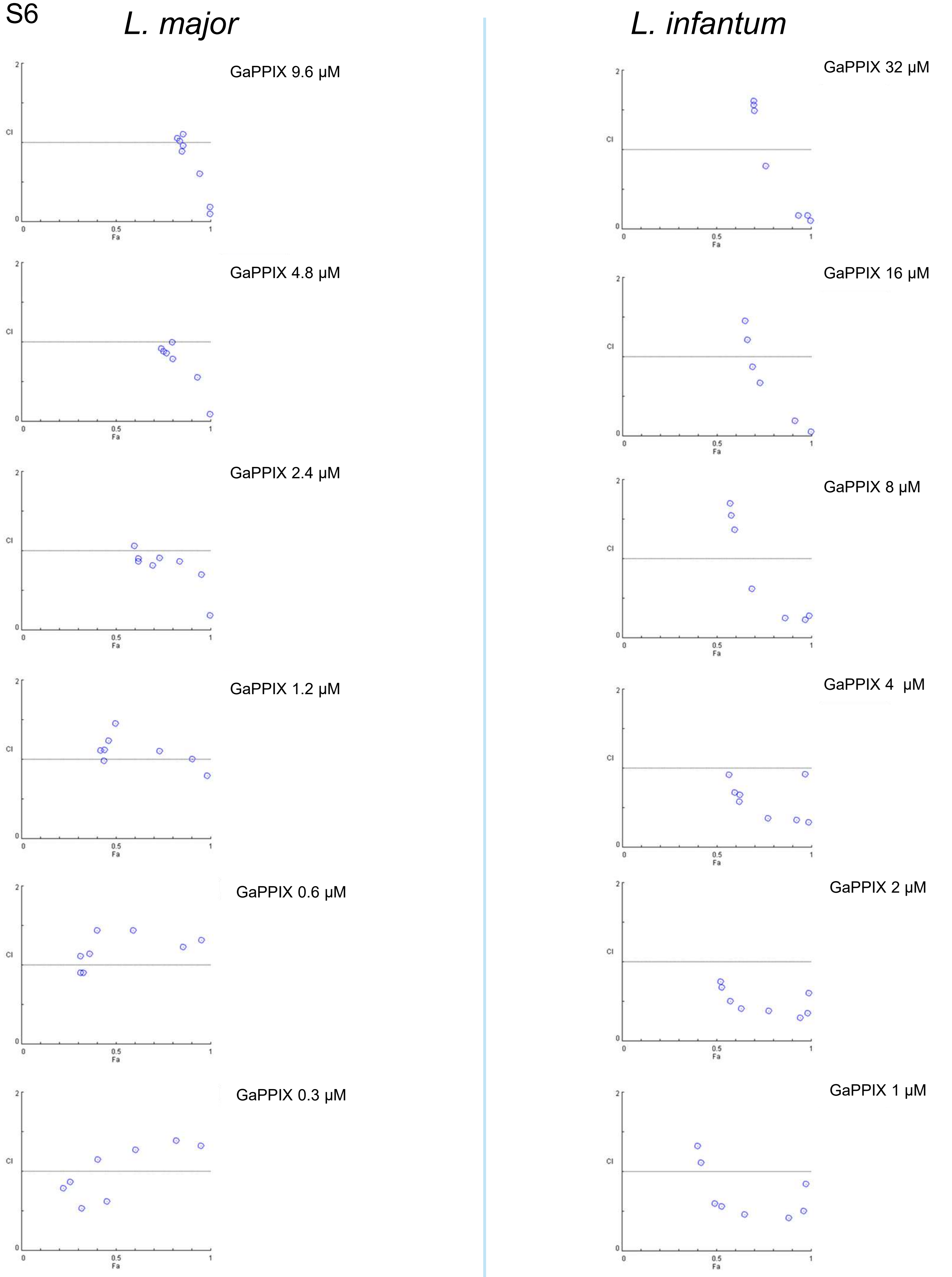

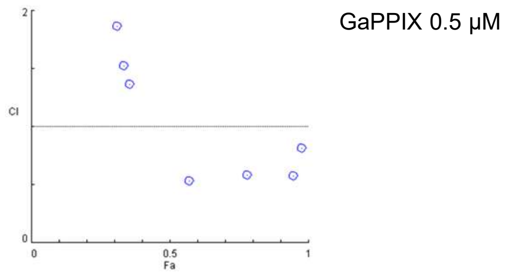

Fig S6. Multiple isobolograms generated using combination indexes (CI) values and the corresponding effect (Fa) for each combination of co-treatment with miltefosine and GaPPIX tested in *L. major* and *L. infantum*, respectively. All combinations tested for *L. major* and *L. infantum* are listed in Table 2 and 3, respectively. The different GaPPIX concentrations used are indicated in each corresponding isobologram. Each point represents a specific co-treatment combination of miltefosine and GaPPIX. Specifically, points with CI < 0.9 indicate combinations resulting in a synergistic interaction; points in the proximity of the dotted line (0.9 < CI < 1.10) suggest combinations having an additive effect and points with a CI > 1.1 indicates combinations with an antagonistic effect.
